# Ultra-portable, wireless smartphone spectrometer for rapid, non-destructive testing of fruit ripeness

**DOI:** 10.1038/srep32504

**Published:** 2016-09-08

**Authors:** Anshuman J. Das, Akshat Wahi, Ishan Kothari, Ramesh Raskar

**Affiliations:** 1 MIT Media Lab, Massachusetts Institute of Technology, 75 Amherst, St. Cambridge, MA, 02139, USA; 2 WeSchool, Lakhamsi Nappu Road, Matunga East, Mumbai, Maharashtra 400019, India; 3 VIT, Near Katpadi Rd, Vellore, Tamil Nadu 632014, India

## Abstract

We demonstrate a smartphone based spectrometer design that is standalone and supported on a wireless platform. The device is inherently low-cost and the power consumption is minimal making it portable to carry out a range of studies in the field. All essential components of the device like the light source, spectrometer, filters, microcontroller and wireless circuits have been assembled in a housing of dimensions 88 mm × 37 mm × 22 mm and the entire device weighs 48 g. The resolution of the spectrometer is 15 nm, delivering accurate and repeatable measurements. The device has a dedicated app interface on the smartphone to communicate, receive, plot and analyze spectral data. The performance of the smartphone spectrometer is comparable to existing bench-top spectrometers in terms of stability and wavelength resolution. Validations of the device were carried out by demonstrating non-destructive ripeness testing in fruit samples. Ultra-Violet (UV) fluorescence from Chlorophyll present in the skin was measured across various apple varieties during the ripening process and correlated with destructive firmness tests. A satisfactory agreement was observed between ripeness and fluorescence signals. This demonstration is a step towards possible consumer, bio-sensing and diagnostic applications that can be carried out in a rapid manner.

Optical spectroscopy has been a very powerful tool in research and industrial applications. It is extensively used in diagnostics^1^,^2^, assessing food quality^3^, environmental sensing^4^, pharmaceutical testing^5^ among several other applications. It has the advantages of being rapid and non-destructive making it a useful tool for qualitative as well as quantitative analysis. However, most spectrometers utilized in the industrial or laboratory based applications are expensive, bulky and require an accompanying computing device to capture data. As a result, many spectroscopic studies are limited to the controlled laboratory settings due to these constraints. Recently, due to advancements in electronics and fabrication methods, portable spectrometers have been realized. Although, these spectrometers are commercially available they do not support standalone operation. Typically, they rely on the use of an external computer to operate, collect and analyze data. This feature significantly increases the cost of operation and limits the range of applications. This limitation led to the use of readily available computing devices like smartphones as interfacing tools with spectrometers. Although, there has been some work on portable smartphone spectroscopy, there is a need for several improvements in terms of quality and repeatability of data^6^,^7^. Typically a grating is used as a dispersing element and the sensor of a cell phone camera is used as the detector^8^. It is to be noted that neither the dispersing element nor the detector is calibrated in a rigorous manner leading to unreliable data. For instance, Bayer filters in the camera sensors have substantial color overlaps making precise spectral measurements challenging. Decoupling of these overlaps is necessary to making accurate measurements which often results in increased computational complexity. Additionally, the cameras on smartphones have automatic adjustments for focus and white balance making it difficult to obtain consistent readings^7^. There have been several reports attempting to characterize the camera sensor to carry out reliable measurements^6^,^7^. Another downside of using the camera as a spectral sensor is that it is unavailable for imaging. In cases where simultaneous imaging and spectral measurements are necessary, this can be a limiting factor. With these limitations, the spectrometer arrangement is more suited for educational or informational purposes rather than a research or measurement tool^8^,^9^.

With recent advancements in materials and fabrication techniques like nanoimprint and MEMS based lithography, compact microcontrollers and wireless technology, it has become possible to realize portable sensing systems on smartphone platforms^10^,^11^,^12^,^13^,^14^,^15^,^16^,^17^,^18^. There have been reports of miniature quantum dot spectrometers, however, no smartphone based demonstration was reported^19^,^20^. We demonstrate for the first time a compact, standalone spectrometer that works with a smartphone via wireless connection. The device is equipped with a software app that interfaces, collects, stores and analyzes data. It is low power and compatible with a range of analog to digital (A/D) converters and smartphones. We present a complete demonstration of a research grade device that can be used for accurate measurements in the lab and field.

We utilize the spectrometer prototype to study UV fluorescence of chlorophyll (ChlF) found in plant based components like fruits^21^. There have been several reports of studying ChlF in a variety of fruits like apples^22^,^23^,^24^, banana^25^,^26^, oranges^27^ among others. ChlF is a good indicator of photosynthetic activity and has been observed to relate to defects^28^, damage^29^,^30^, senescence and ripening of post harvest fruits^31^,^32^. For instance, researchers used ChlF in bananas to study cell death and observed fluorescent halos around black spots on the surface of the fruit^26^. Apart from ripening and senescence, ChlF has also been used to study flavonoids and anthocyanins in fruits^33^,^34^,^35^. One particular advantage of measuring ChlF in fruits is that it can be used to detect fruit ripeness in a non-destructive manner. Most of the earlier reports demonstrate spectroscopic applications in laboratory settings and have indicated the advantages of using compact devices for field studies. The current report addresses this issue and complements the progress in the field of portable spectroscopy. In this report, we utilized the smartphone spectrometer to rapidly evaluate ripeness of different varieties of apples using ChlF emissions when excited using UV light. We observe a satisfactory correlation in the ripeness measured by a penetrometer and ChlF measured using our device.

## Results

**Figure 1:**
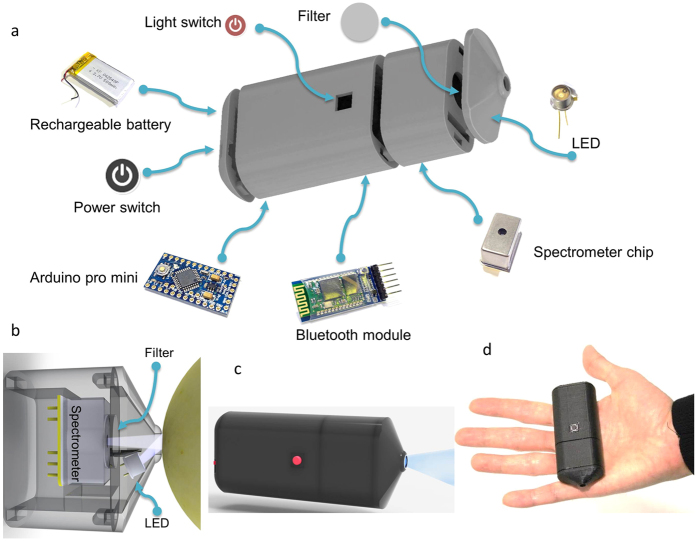
(**a**) Schematic of the different components of the smartphone spectrometer prototype. (**b**) Close-up of the nozzle depicting illumination and collection geometry. (**c**) Model of the assembled prototype. (**d**) Photograph of a working prototype.

A schematic of the various components of the prototype is shown in Fig. 1(a). The assembly consists of the spectrometer chip, white or UV LED, optical filters, a Bluetooth module for wireless data transfer, a microcontroller for A/D conversion and clock generation, a rechargeable Li-ion battery along with switches for power and the light source. The outer casing was 3D printed and the device dimensions were 88 mm × 37 mm × 22 mm as shown in Fig. 1(b–d). The LED was arranged in the vicinity of the nozzle opening at an angle to the spectrometer allowing efficient illumination with a spot size of 4.5 mm as shown in Fig. 1(b). Analog signal from the spectrometer consisted of a train of pulses whose temporal position in the pulse train was correlated to the spatial position of the pixel. The linear sensor within the spectrometer chip had 256 pixels corresponding to wavelengths in the range of 340–780 nm. The sensor required a clock pulse (CLK) for its operation which was set to 1 kHz in the current setup. A start pulse (ST) was used to trigger the pixel readout process and the interval between two consecutive ST pulses was the integration time of the sensor. On receiving the command from the companion application to capture the spectrum, a ST pulse was triggered which initiated the charge integration of each pixel. After the required integration time a second ST pulse was triggered to end the charge integration. Considering a CLK of 1 kHz and video output frequency which is 1/4 of the clock frequency (250 Hz), it would take about 1.024 s (4/1000 × 256 pixels) to complete acquisition of all pixels as shown in the timing diagrams in Supplementary Fig. S1. Since the readout is sequential, charge integration occurs at different times on each pixel and the next start pulse can be generated only after the readout is completed. The sensor also provided an end of scan (EOS) signal which was used to terminate the read out process and begin the data transmission process via Bluetooth to any smartphone which was equipped with the companion application.

**Figure 2:**
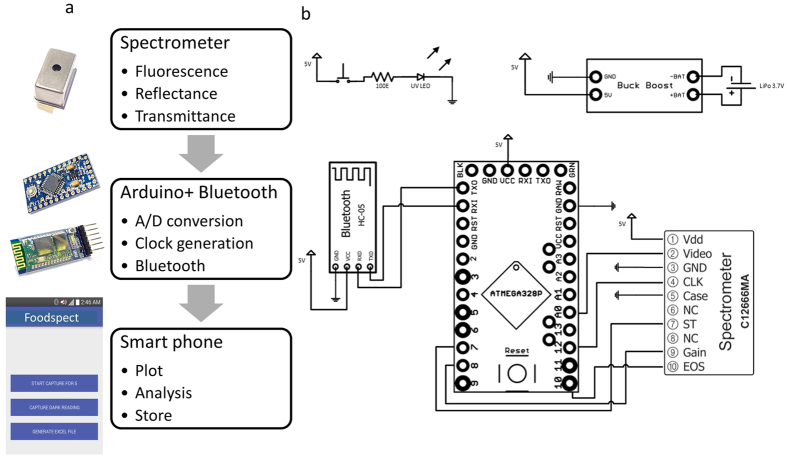
(**a**) Integration scheme of the smartphone spectrometer. (**b**) Circuit diagram of the prototype indicating the various components involved- spectrometer chip, microcontroller, Bluetooth interface and battery connections.

**Figure 3:**
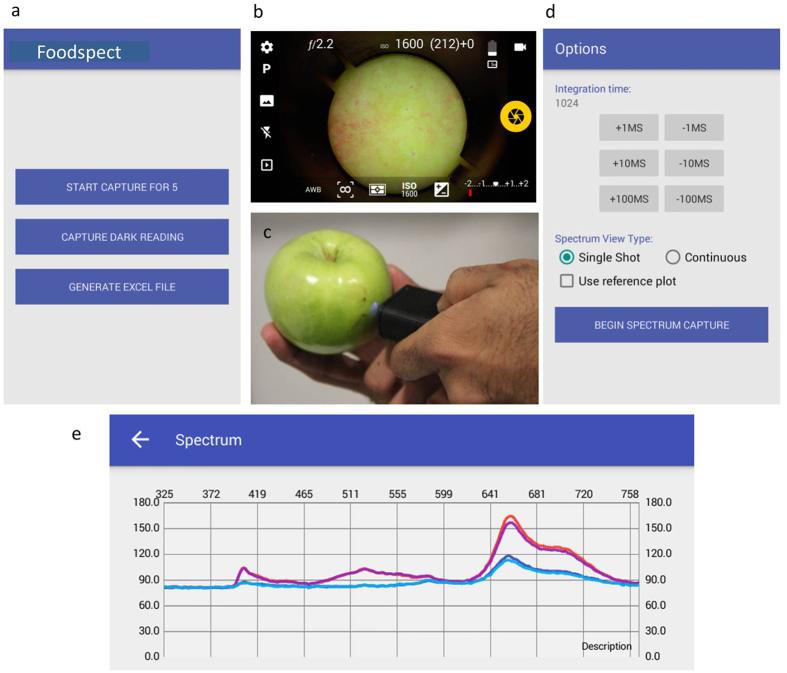
Process of data acquisition using the app interface on the smartphone. (**a**) Starting interface on the app with a options menu. (**b**) Typical image of an apple surface captured by the smartphone magnifier (**c**) Photograph of the process of spectral data acquisition. (**d**) Interface for spectral acquisition with integration times and modes of operation (**e**) Typical ChlF for Golden Delicious apple sample.

Once the pixels were correlated to the pulse train a calibration step was performed to convert pixels to wavelength. A calibration equation was applied to the pixel information to perform this conversion. A 5^*th*^ order polynomial was used to fit the data and extract wavelength information. Please refer to Methods and Supplementary Table T1 for more details. A step by step protocol for the integration process is shown in Fig. 2(a,b). Subsequent to the calibration a Bluetooth interface was setup to communicate with the smartphone. A customized app was developed on the Android operating system to communicate with the spectrometer assembly, plot and analyze the spectra on the smartphone. Operation protocol of the app is depicted in Fig. 3. Please refer to the Methods section for details.

### Performance comparison with commercial spectrometers

**Figure 4:**
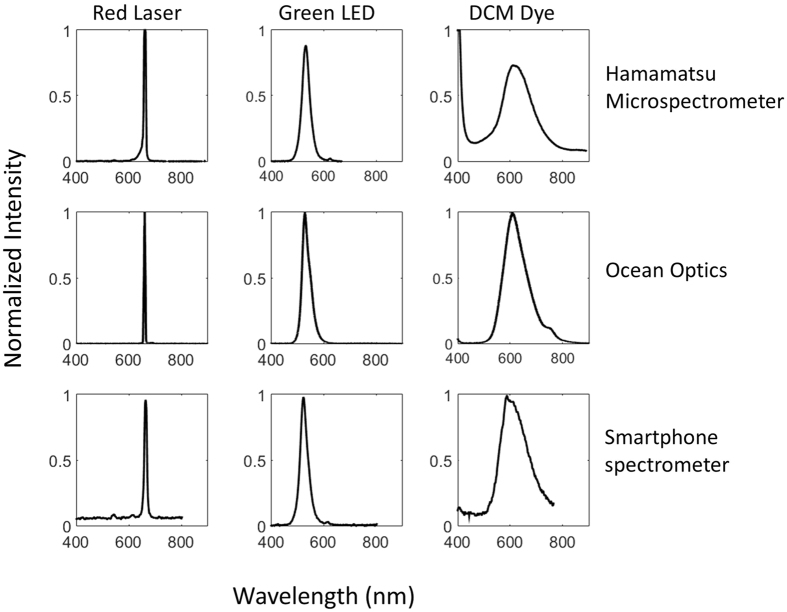
Performance comparison of smartphone spectrometer with Ocean and Hamamatsu USB spectrometers for different emission sources- red laser, green LED and fluorescence from DCM dye. First row, normalized spectra captured using a USB based Hamamatsu microspectrometer. Second row, normalized spectra obtained using Ocean Optics spectrometer. Third row, normalized spectra captured using the smartphone spectrometer.

Spectral data from our prototype was compared to commercial spectrometers like Ocean Optics (USB4000) and Hamamatsu Micro-spectrometers with universal serial bus (USB) based interface circuits. This process was beneficial not only to compare the accuracy of the device but also provided a rigorous calibration step. Emission and fluorescence spectra from various sources were measured using different spectrometers and a strong correlation was observed in the spectral characteristics of the signal. Three sources with varying spectral bandwidths were used as tests for the comparison study; a) a narrowband (Δ*λ* = 7 nm) laser diode with emission centered at 660 nm, b) A green LED with intermediate emission bandwidth (Δ*λ* = 35 nm) and (c) a laser dye with broadband emission (Δ*λ* = 100 nm). The laser dye used in this study 4-(Dicyanomethylene)-2-methyl-6-(4-dimethylaminostyryl)-4H-pyran (DCM) was illuminated with a UV source and the fluorescence was captured using the various spectrometers. As shown in Fig. 4, the smartphone spectrometer exhibited a strong correlation across the different sources in terms of center emission wavelength and spectral width as compared to commercial spectrometers. A summary of the performance of the smartphone spectrometer is presented in Table 1 along with a comparison with commercially available spectrometers. The cost of this prototype is significantly lower than commercial spectrometers with similar performance features. Currently, smartphones can be procured for about $50 making the entire assembly under $250.

### UV fluorescence of Chlorophyll for non-destructive ripeness testing

In order to verify the working of our device we carried out ChlF detection in a variety of apple (*Malus domestica*) samples. There have been numerous studies indicating that ripeness of fruits can be correlated to ChlF. We carried out ripeness estimation using mechanical firmness testing and compared the data with ChlF measured from our device. The peak intensity of ChlF emission at 680 nm (*F*_*R*_) was related to photosynthetic activity and ripening. We observed ChlF signals from a variety of apple types based on color- homogeneous green or yellow, mixed color and homogeneous red apple samples, although the intensity varied from the apple type.

#### Homogeneous Green/Yellow apples

**Figure 5:**
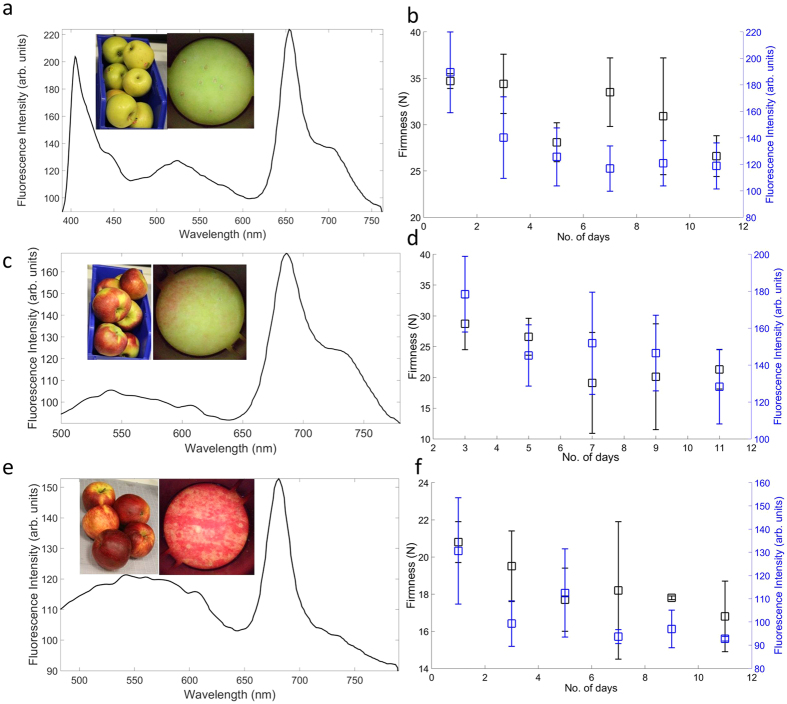
Results of fluorescence measurements from the three apple varieties. Typical fluorescence spectrum for various samples (**a**) Golden delicious, (**c**) McIntosh and (**e**) Empire, respectively. Evolution of firmness (black squares, left y-axis) ChlF signal (blue squares, right y-axis) over time for the varieties (**b**) Golden delicious, (**d**) McIntosh and (**f**) Empire, respectively. (Insets) Photographs of the samples along with a magnified image of the region that was tested.

These varieties exhibited the strongest fluorescence signal (*F*_*R*_ > 200 counts) due to larger concentrations of chlorophyll in the skin of the apple. Variety that was studied in this category was Golden Delicious. The skin color in this variety was uniform all over the sample which made the selection of the region of interest straightforward. Figure 5(a,b) shows a typical ChlF plot along with time evolution of ChlF for this variety. An intense ChlF signal was observed at 680 nm and a relatively lower intensity signal at 730 nm as expected. The shape and position of the peaks is consistent with earlier studies of the ChlF signal. A downward trend was observed for both the fluorescence intensity and the firmness indicating the loss of chlorophyll fluorescence was related to the ripening of the fruit. A linear fit was performed for the fluorescence signal as a function of time and the normalized residuals resulted in a *R*^2^ value of 0.65 indicating a good fit. This variety also exhibited appreciable fluorescence (*F*_*R*_ ≈ 120 counts) in the 500–600 nm wavelength range which can be attributed to the presence of phenolic compounds like carotenoids in the skin^36^. Although it is speculated that the fluorophores responsible for this emission are present in the mesophyll of leaves, the exact reason of this emission remains to be investigated^36^.

#### Mixed color apples

These varieties are characterized by mixed regions of red and green/yellow on the surface. Green/yellow regions exhibited significantly larger (200 > *F*_*R*_ > 150 counts) ChlF signal as compared to the red regions (*F*_*R*_ < 150 counts). These high ChlF sites were consistently found in all the samples studied and were used for fluorescence measurements. The variety that was studied in this category was McIntosh. Figure 5(c,d) shows a typical fluorescence signal for this variety along with a time evolution of the ChlF signal. A *R*^2^ value of 0.74 was observed in the ChlF time evolution measurements indicating that the device works well for these varieties. A small fluorescence signal (*F*_*R*_ ≈ 100 counts) was observed in the 500–600 nm wavelength range for this variety and the source of this emission is similar to the case of green apples.

#### Homogeneous Red apples

This variety comprises of samples with predominantly red coverage on the surface. The fluorescence signal is the weakest as compared to the mixed and green-yellow varieties. This is due to the fact that anthocyanins present in the skins of these varieties partially mask the ChlF signal^34^. There have been earlier reports of characterizing them using additional features along with ChlF^22^. Figure 5(e,f) shows a typical fluorescence spectrum for this variety. Linear fit on the Chlf time evolution data yielded an *R*^2^ of 0.61. This variety also exhibited a sizable blue-green fluorescence signal in the 500–600 nm spectral range (*F*_*R*_ ≈ 120 counts) which may be attributed to the presence of phenolic compounds present in the skin.

## Discussion

Smartphone spectroscopy opens up several avenues for field testing that were not feasible earlier. Several factors had contributed to the limited use of spectrometers in the field and consumer applications. Although size and cost are the more obvious factors other factors like stray light and reliability have contributed to challenges. With the availability of MEMS based spectrometers the challenge of size has been addressed. As can be seen in Fig. 1 and Table 1, the entire prototype weighs 48 g and dimensions are 88 mm × 37 mm × 22 mm. Rapid evolution of electronics and the demand has addressed the cost aspect and the spectrometer chip can be procured for about $180 and the entire assembly along with the smartphone can be realized under $250. Stray light can be a source of noise in spectroscopic measurements and as a result, most reports demonstrate experiments in controlled laboratory settings. Our device overcomes this challenge by launching and collecting light through a nozzle-like enclosure, thereby shielding any stray light contribution as shown in Fig. 1(b). This feature makes it attractive for field based applications. A limiting factor in the current setup is the relatively low ADC bit resolution. Although the wavelength resolution is comparable to the commercial spectrometers used in this study, the ADC resolution in the microcontroller may limit some studies where small intensity changes need to be studied. The sensitivity of the spectrometer depends on several factors like the optical system efficiency, diffraction efficiency of the grating, sensitivity of the image sensor, charge-to-voltage converter constant and the A/D converter resolution. While the other parameters remain unchanged in the process of integration, the A/D converter resolution is affected by the choice of microcontroller. In the current device the bit depth is 10 bit as compared to commercial spectrometers (16 bit) which leads to a lower sensitivity. This limiting factor is due to the unavailability of compact microcontrollers with higher bit depth. However, with rapid progress in this field, the availability of better microcontrollers can be anticipated in the near future.

Smartphone based fruit testing can be beneficial for several end-users. Currently, testing of ripeness in apples and several other fruits is carried out in a destructive manner using mechanical tests. Optical tests are non-destructive and can assist farmers in determining optimum harvest times. The device can be used for rapid sorting in storage facilities for different varieties of fruits and to assess ripening. Furthermore, the device could be adapted for consumer applications for testing of fruit ripeness and screening for defects that may not be visually captured. *R*^2^ values from the time evolution studies are comparable with previous reports^22^ and can be improved by carrying out studies with a large number of samples. Studies can be expanded to several other fruits and vegetables where ChlF has shown to be beneficial in quantifying properties like stress and damage. Additionally, the device could be used to study ChlF in leaves and operate in the reflectance or transmittance mode for several other applications.

The portable nature of the device along with simplified data collection methods can have a huge advantage in gathering large sets of data that may be useful in building machine learning based models. The output of these models can be used to accurately determine the right harvest times or find uses identification and sorting at a large scale.

## Methods

### Spectrometer Characteristics

A Hamamatsu micro-spectrometer (C12666MA) sensor was obtained from Hamamatsu Photonics and the sensor was detached from the assembly. The analog video output from the spectrometer was used to connect to an Arduino microcontroller. The pin configurations of the Arduino and the spectrometer have been provided in Fig. 2. The output of the Arduino was connected to the Bluetooth chip and the data was transmitted to the phone via the phone Bluetooth. The spectrometer was fabricated using MEMS technologies with a curved grating and a slit with length of 500 *μm* and a width of 50 *μm*. The wavelength sensitivity was in the range of 340–780 nm. Data was collected from the analog pin of the spectrometer and digitized using an Arduino pro mini with a bit resolution of 10 bits. The spectrometer had a spectral resolution of 15 nm and integration times in the range of ms-s with possibility of external triggering.

### Smartphone Integration and calibration

The prototype comprised of a Bluetooth enabled smartphone and Bluetooth module interfaced with the A/D conversion Arduino chip. Data (pixels values) are transmitted via Bluetooth configured as Serial Port Profile over a baud rate of 9600 bits per second. Data was transmitted in a raw A/D format against the pixel number. Calibration was carried out using a 5^*th*^ order polynomial equation of the form,


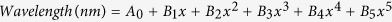


where, *x* is the pixel number and *A*_0_, *B*_1_, *B*_2_, *B*_3_, *B*_4_, *B*_5_ are calibration coefficients whose values are presented in Supplementary Table T1.

### Prototyping

Computed aided design and 3D printing were used to prototype the enclosure to house the battery, spectrometer and Bluetooth communication chip. The nozzle like structure was used to direct and receive light as shown in Fig. 1.

### Light Source and filter

UV LED with a wavelength of 360–380 nm was used as an excitation source. A nominal current of 15 mA was supplied under a bias of 3.6 V using a rechargeable Li-ion battery. UV long pass filter was used with a cut-off wavelength of 400 nm.

### Characteristics of smartphone app

An in-house developed app was used to interface and capture the data. The app had all necessary features like dark subtraction, control over integration time, reference plots and was capable of capturing, plotting and exporting data. First, a sample number was pre-allotted and a dark reading was captured. Second, an image of the region of interest was captured using a cross-polarizer and lighting arrangement on the smartphone camera. This was used to suppress specular surface reflections from the fruit surface. Third, the spectrometer was pointed to the region of interest which was imaged earlier and ChlF spectra were obtained at those locations. The data was then saved on the smartphone for further processing.

### Performance Comparison

Commercial spectrometer systems like Ocean Optics USB4000 and Hamamatsu Micro-spectrometer (C12880MA) with USB Board (C13016) were compared to the smartphone spectrometer. DCM dye was dissolved in acetone/PMMA mixture and drop casted on to a glass substrate. A UV LED with wavelength of 380 nm was used to excite the dye and the fluorescence signal was captured using the three devices. A commercially available red laser and a green LED were also used for performance comparison.

### Setup for testing and sample preparation

Fruit samples were cleaned and several regions were marked before the measurements. A high resolution image of the region was captured using a polarized smartphone magnifier. Subsequently, the selected region was excited by a UV LED and the emitted fluorescence was captured in the smartphone spectrometer. The primary emitter in the case of fruits is chlorophyll and its fluorescence was recorded on the smartphone. After the fluorescence measurements were collected, the same region was analyzed for firmness using a digital penetrometer (FHT 1122) with a probing head of 7.9 mm diameter. The region was peeled and the penetrometer was inserted into the pulp until a designated depth and the firmness reading was noted. The samples that were studied in this report are Golden Delicious, Empire and McIntosh apples. This process was repeated over a time frame of 11 days for all the varieties that were studied.

### Data Analysis

Spectral data was extracted from the smartphone to carry out further analysis of fruit ripening. Every fruit sample was imaged at two locations and the firmness data along with spectral data was averaged. A common background noise signal was subtracted and peak intensity of ChlF at 680 nm was extracted. The strength of this signal was tracked over the duration of the study.

## Additional Information

**How to cite this article**: Das, A. J. *et al*. Ultra-portable, wireless smartphone spectrometer for rapid, non-destructive testing of fruit ripeness. *Sci. Rep.* **6**, 32504; doi: 10.1038/srep32504 (2016).

## Supplementary Material

Supplementary Information

## Figures and Tables

**Table 1:** Performance comparison of commercial spectrometers with the smartphone spectrometer.

Specification	Ocean Optics	Hamamatsu with USB Board	Smartphone spectrometer with Hamamatsu Sensor
Spectral Resolution	10 nm	15 nm	15 nm
Bandwidth	360–1100 nm	340–840 nm	340–780 nm
ADC Bits	16	16	10
Operating System	Windows	Windows	Android
Size	89.1 mm × 63.3 mm × 34.4 mm	108 mm × 70 mm × 35 mm	88 mm × 37 mm × 22 mm
Weight	224 g (without light source)	198 g (with light source and battery, built in-house)	48 g (with light source, battery, Bluetooth connection, microcontroller, filter)
Cost	$4000	$1200	under $250 (including smartphone)
