# Supplementary material for: Ultra-portable, wireless smartphone spectrometer for rapid, non-destructive testing of fruit ripeness
Source: Sci Rep. 2016 Sep 8;6:32504. doi: 10.1038/srep32504 (PMC5015488; doi:10.1038/srep32504)
Supplement: Supplementary Information [file srep32504-s1.pdf]

Ultra-portable, wireless smartphone spectrometer for rapid, non-destructive testing of fruit ripeness

Anshuman J. Das, Akshat Wahi, Ishan Kothari and Ramesh Raskar

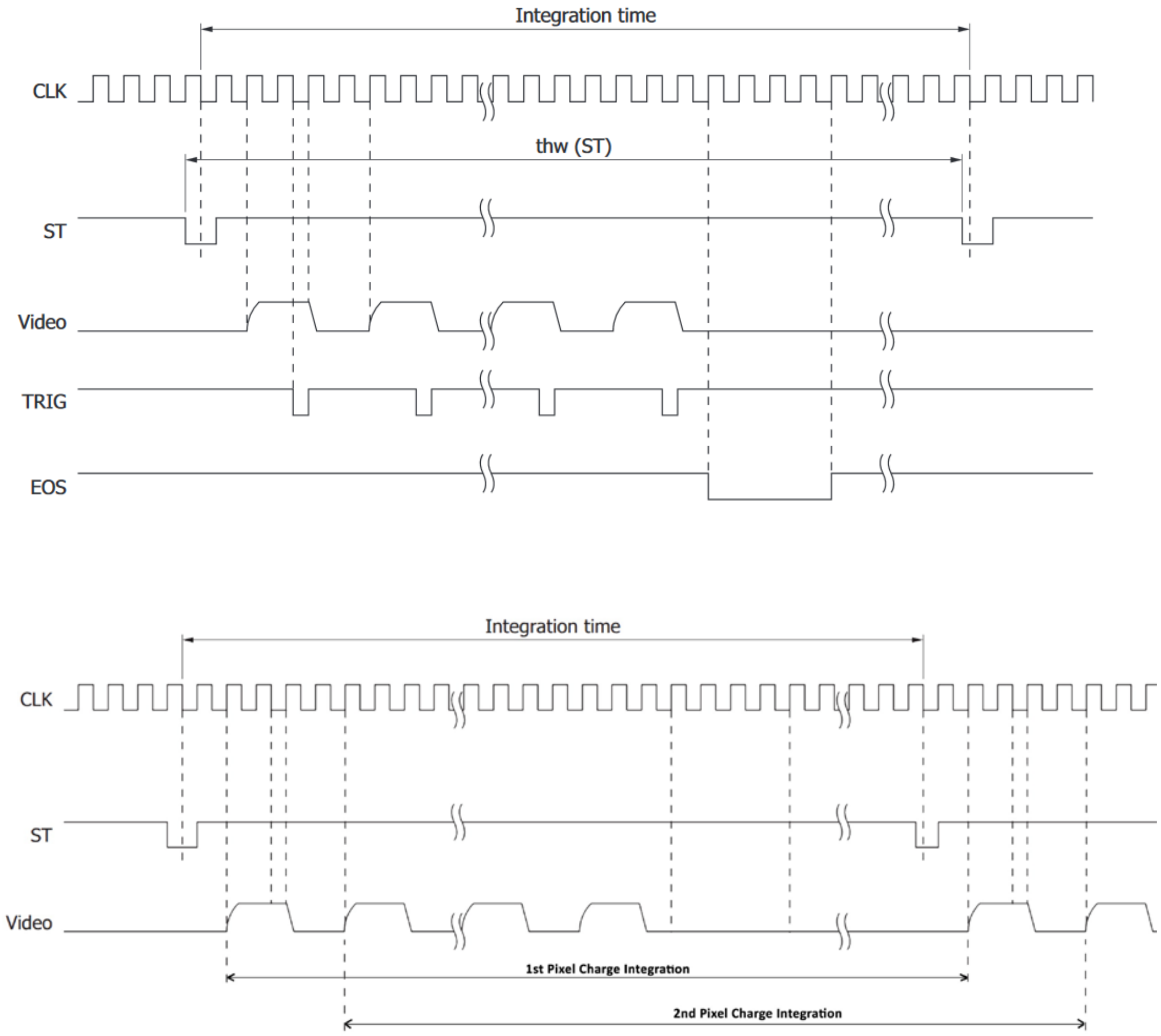

Supplementary Fig. S1: Timing diagram of the spectrometer showing the various signal involved in the operation.

(Adapted from- http://www.hamamatsu.com/resources/pdf/ssd/c12666ma_kacc1216e.pdf)

Supplementary Table T1: Calibration coefficients for the spectrometer

Coefficient	Value
A0	2.988204530e+002
B1	2.402512393e+000
B2	-9.234466848e-004
B3	-4.648308454e-006
B4	2.090258796e-010
B5	1.496304653e-011

Note: These coefficients vary for every spectrometer chip. The actual coefficients can be found in the test sheet accompanying the product.
